# Association between annual change in FEV_1_ and comorbidities or impulse oscillometry in chronic obstructive pulmonary disease

**DOI:** 10.1186/s12890-022-01980-6

**Published:** 2022-05-08

**Authors:** Hiroyuki Sugawara, Atsushi Saito, Saori Yokoyama, Kazunori Tsunematsu, Hirofumi Chiba

**Affiliations:** 1 Sugawara Internal Medicine and Respiratory Clinic, Tomakomai, 053-0821 Japan; 2 Department of Respiratory Medicine and Allergology, Sapporo Medical University School of Medicine, S1W16 Chuoku Sapporo, Hokkaido, 060-8543 Japan

**Keywords:** Chronic obstructive pulmonary disease, Impulse oscillometry system, Pulmonary function test, COPD Assessment Test, St. George’s Respiratory Questionnaire

## Abstract

**Background:**

Chronic obstructive pulmonary disease (COPD) is characterized by persistent respiratory symptoms and airflow limitation. The decline in forced expiratory volume in one second (FEV_1_) is considered to be one of the most important outcome measures for evaluating disease progression. However, the only intervention proven to improve COPD prognosis is smoking cessation. This study therefore investigated the factors associated with annual FEV_1_ decline in COPD.

**Methods:**

This retrospective study followed up 65 patients treated for COPD for 5 years: 13 current smokers and 52 former smokers, 25 with pneumonia, 24 with asthma, 18 with cancer, and 17 with cardiovascular disease. The patients were divided into groups based on clinical cutoff parameters of the impulse oscillometry system (IOS): 11 high and 54 low R5, 8 high and 57 low R20, 21 high and 44 low R5–R20, 26 high and 39 low X5, 38 high and 27 low Fres, and 36 high and 29 low AX. We investigated whether the decline in FEV_1_ was associated with comorbidities and IOS parameters.

**Results:**

The annual change in FEV_1_ over 5 years was significantly affected by smoking status (current − 66.2 mL/year vs. former − 5.7 mL/year, *p* < 0.01), pneumonia (with − 31.5 mL/year vs. without − 8.9 mL/year, *p* < 0.05), asthma (with − 30.2 mL/year vs. − 10.8 mL/year, *p* < 0.01), but not by cancer and cardiovascular disease. In the groups defined by IOS results, only the high AX group had significantly more annual decline in FEV_1_ and %FEV_1_ than the low AX group (− 22.1 vs. − 12.8, *p* < 0.05 and − 0.20 vs. 0.40, *p* < 0.05, respectively).

**Conclusions:**

Continuing smoking as well as complications in pneumonia and asthma would be risk factors for the progression of COPD. AX might be a suitable parameter to predict the prognosis of patients with COPD.

**Supplementary Information:**

The online version contains supplementary material available at 10.1186/s12890-022-01980-6.

## Background

Chronic obstructive pulmonary disease (COPD), which is characterized by persistent respiratory symptoms and airflow limitation caused by a mixture of small airway disease and parenchymal destruction, has become one of the top three causes of death worldwide and is a major cause of chronic morbidity and mortality [1]. To evaluate disease progression, one of the most important outcome biomarkers is the decline in forced expiratory volume in one second (FEV_1_) [2–5]. Cigarette smoking is well known as the most common risk factor for COPD; smokers have a higher prevalence of respiratory symptoms and abnormalities of respiratory function, a greater annual rate of decline in FEV_1_, and higher mortality than non-smokers [6]. Smoking cessation is the only intervention that has conclusively been shown to alter the rate of decline in FEV_1_ [7]. Long acting muscarinic antagonist (LAMA) and long acting β2 agonist (LABA) are now used for the treatment of COPD, but there are few data assessing the effects of smoking cessation in patients taking these drugs.

COPD often coexists with other diseases (comorbidities), such as pneumonia, asthma, cancer, and cardiovascular disease, that may have a significant impact on prognosis [8], but the impact of these comorbidities on FEV_1_ decline requires further investigation. Exacerbations have negative impacts on respiratory function and lead to worsening of the chronic progressive course of this disease [9], and pneumonia is a known cause of exacerbation, but few studies have investigated the relationship between episodes of pneumonia and annual decline of FEV_1_ in COPD. The prognosis of ACO (asthma-COPD overlap) patients is controversial [10–12]. Cancer has a great impact on the quality of life (QOL) and prognosis of COPD patients [13], and reduction in FEV_1_ is strongly associated with a higher incidence of atrial fibrillation and heart failure [14], but there have been few studies on how much cancer and cardiovascular disease affect respiratory function.

The impulse oscillometry system (IOS) can be used to assess the function of large and small airways. It is a noninvasive device which assesses respiratory function by the forced oscillation technique [15–17]. Our previous work revealed that phenotypic difference in IOS parameters could be associated with the efficacy of inhaled corticosteroids (ICS) in asthma and cough variant asthma [18, 19]. We therefore hypothesized that in COPD, IOS may have the potential to detect airway obstruction earlier than spirometry.

The aim of this study is to clarify how the rate of FEV_1_ decline is affected by smoking and comorbidities such as pneumonia and asthma, and to investigate whether baseline IOS parameters could predict the future decline of respiratory function in patients with COPD.

## Methods

### Participants and treatment

This was a single-center retrospective observational study. Patients with COPD treated in the clinic between January 2012 and December 2015 were included in the study. All patients presented with chronic dyspnea, chronic cough or sputum production, and a history of smoking, and were diagnosed by confirming the presence of persistent airflow limitation based on post-bronchodilator FEV_1_/forced vital capacity (FVC) < 0.70 [20]. Patients were assessed by spirometry and IOS and followed up for 5 years. Patients were excluded if treatment was interrupted during the follow-up period or if they were transferred to another hospital.

At the first visit, demographic information, including gender, age, height, weight, smoking history, medical history and any medications, and information on pulmonary symptoms (modified Medical Research Council [mMRC] Dyspnea Scale and COPD Assessment Test [CAT]), and comorbidities were collected.

Patients diagnosed with COPD were treated with LAMA, LABA, and added ICS if they had an asthma phenotype, according to the Global Initiative for Chronic Obstructive Lung Disease (GOLD) guidelines [21]. This study was conducted in accordance with the declaration of Helsinki and was approved by the institutional ethical committee of Sapporo Medical Association. The experimental protocols and the purpose of the research were explained to all participants and informed consent was obtained in the form of opt-out on the website.

### Measurements of IOS and respiratory function

IOS was measured using a commercially available impulse oscillometry device (MasterScreen IOS, Jaeger, Germany) according to the manufacturer’s recommendations [17]. The resistance at 5 Hz (R5: indicating total airway resistance), resistance at 20 Hz (R20: representing central airway resistance), difference between R5 and R20 (R5 − R20: index of the small airways), reactance at 5 Hz (X5: relating to compliance), resonant frequency (Fres), and integrated area of low frequency X (AX) were evaluated [22–24]. The use of Fres and AX has been proposed to detect the degree of obstruction in the peripheral airways [15, 22, 25].

After measuring IOS, a pulmonary function test was performed using spirometry (MasterScreen IOS, Jaeger, Germany). The tests were performed in this order to prevent any negative effects of forced expiration on the airway. The percentage predicted forced vital capacity (%FVC), percentage predicted forced expiratory volume in one second (%FEV_1_), FEV_1_/FVC ratio, percentage predicted maximal mid-expiratory flow (%MMEF), and percentage predicted peak expiratory flow (%PEF) were assessed.

For parameters of IOS, only a few predicted values are available for Caucasians according to Vogel & Smidt [26] and there are no defined reference values for COPD. We adopted cutoff IOS values for COPD based on previous reports [25, 27–31], as follows: R5 = 0.39, R20 = 0.27, R5–R20 = 0.10, X5 =  − 0.13, Fres = 17.7, and AX = 0.55. If the measured value was higher or lower than the cutoff value, it was recorded as a high or low IOS parameter, respectively.

### Assessment of dyspnea and QOL

We assessed the two most widely used measures of symptoms according to GOLD [8]. Dyspnea was evaluated according to the modified British Medical Research Council (mMRC) scale. A questionnaire scale was considered adequate to assess symptoms. The mMRC is a grading scale from 0–4 which relates well to other measurements of health status [32].

The COPD Assessment Test (CAT™) is an eight-item unidimensional measure of health status impairment in COPD [33]. This score ranges from 0–40 and correlates closely with St. George’s Respiratory Questionnaire (SGRQ) [34].

### Retrospective observation of annual change in FEV_1_

For patients who were current smokers, we explained the need for smoking cessation to patients diagnosed with COPD, instructed them to quit smoking, and provided smoking cessation treatment if necessary. The patients’ respiratory function was retrospectively observed for 5 years. Baseline respiratory function and smoking status were recorded at least 3 months after the start of COPD treatment. Spirometry was performed every 6 months in patients who inhaled LAMA or/and LABA the morning of the test, and the annual change in values and % predicted was calculated.

### Statistical analysis

Numeric variables are expressed as means ± standard error of mean. Differences between two groups were assessed using unpaired two tailed t-tests. Categorical variables were compared using chi-square tests. A *p* value < 0.05 was considered statistically significant. Microsoft Excel 2016 (Microsoft Corporation, the USA), Excel Statistical Program File (ystat2008.xls, Igakutosho-shuppan Ltd., Tokyo, Japan), GraphPad Prism v8 (GraphPad, Inc., San Diego, CA, the USA), and an open-source R statistical software package were used for data analysis and graph generation.

## Results

### Selection of participants

**Fig. 1:**
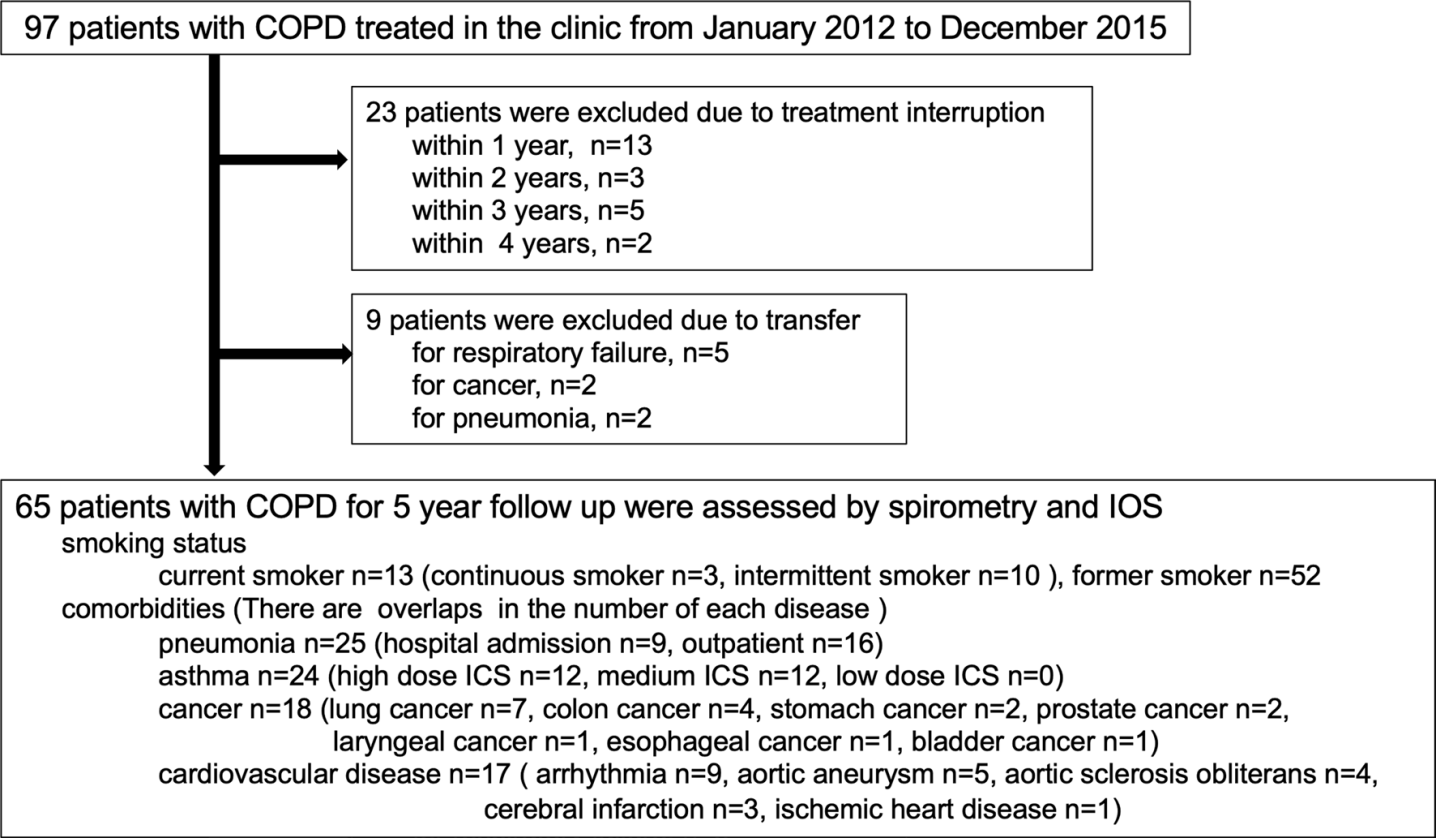
Selection of participants. Of 97 patients diagnosed with COPD who were treated in the clinic between January 2012 and December 2015, 32 patients were excluded and 65 participated in this study

We screened 97 patients with COPD during the inclusion period. Of these, 23 were excluded due to treatment interruption [in < 1 year (*n* = 13), 1 to < 2 years (*n* = 3), 2 to < 3 years (*n* = 5), or in 3 to < 4 years (*n* = 2)] and 9 patients were excluded due to hospital transfer [for respiratory failure (*n* = 5), for cancer (*n* = 2), or for pneumonia (*n* = 2)]. After exclusions, 65 patients with COPD were assessed by spirometry and IOS and followed up for 5 years (Fig. 1).

Smoking status and comorbidities are described in Fig. 1. Patients were current smokers (*n* = 13, comprising 3 continuous smokers and 10 intermittent smokers) or former smokers (*n* = 52, including patients who quit smoking on being diagnosed with COPD). Recorded comorbidities were pneumonia (*n* = 25: hospital admission *n* = 9, outpatient *n* = 16), bronchial asthma (*n* = 24: high dose ICS n = 12, intermediate ICS *n* = 12, low dose ICS *n* = 0), cancer (*n* = 18: lung cancer *n* = 7, colon cancer *n* = 4, stomach cancer *n* = 2, prostate cancer *n* = 2, laryngeal cancer *n* = 1, esophageal cancer *n* = 1, bladder cancer *n* = 1), cardiovascular disease (*n* = 17: arrhythmia *n* = 9, aortic aneurysm *n* = 5, arterial sclerosis obliterans *n* = 4, cerebral infarction *n* = 3, ischemic heart disease *n* = 1). Some patients had more than one disease.

The baseline characteristics of the 65 participants and their spirometry and IOS 5 years after treatment are shown in Table 1. FEV_1_ and FEV_1_/FVC significantly decreased between baseline and 5 years but there were no significant changes in % FEV_1_, % FVC, % MMEF, or %PEF. Except R20, all IOS parameters were significantly reduced at 5 years after treatment.

**Table 1:** Baseline characteristics and comparison of respiratory function between baseline and 5 years after observation in patients with COPD

	Baseline	5 years after
Number of participants	65	65
Age	69.7 (1.0)	
Sex; male: female	53:12	
Body mass index	22.5 (0.4)	21.8 (0.4)
Smoking history (pack-years)	51.7 (1.9)	
Current: former	13: 52	8: 57
mMRC	1.3 (0.1)	1.6 (0.1)**
CAT	6.0 (0.6)	6.9 (0.6)*
*GOLD stage*		
I, n (%)	11 (16.9)	13 (20.0)
II, n (%)	39 (60.0)	35 (53.8)
III, n (%)	14 (21.5)	15 (23.1)
IV, n (%)	1 (1.5)	2 (3.1)
*Spirometry*		
FEV1 (L)	1.66 (0.07)	1.57 (0.08)**
FEV1 (% predicted)	62.8 (2.0)	63.1 (2.2)
FVC (% predicted)	88.7 (1.7)	90.3 (2.0)
FEV1/FVC (%)	56.4 (1.1)	54.8 (1.2)*
MMEF (% predicted)	19.9 (1.2)	19.3 (12)
PEF (% predicted)	68.7 (2.5)	68.1 (3.3)
*IOS*		
R5 (kPa/L/s)	0.29 (0.01)	0.33 (0.02)**
R20 (kPa/L/s)	0.21 (0.01)	0.21 (0.01)
R5-R20 (kPa/L/s)	0.08 (0.01)	0.11 (0.01)**
X5 (kPa/L/s)	− 0.13 (0.01)	− 0.17 (0.02)*
Fres (Hz)	18.7 (0.7)	20.4 (0.8)**
AX (kPa/L)	0.92 (0.11)	1.42 (0.17)**

Data are presented as mean (standard error of mean) or number (percentage). The differences between baseline and 5 years after treatment were evaluated using paired t-tests

^*^p < 0.05

^**^p < 0.01

### ***Annual change in FEV***_***1***_***and IOS in smoking status***

**Fig. 2:**
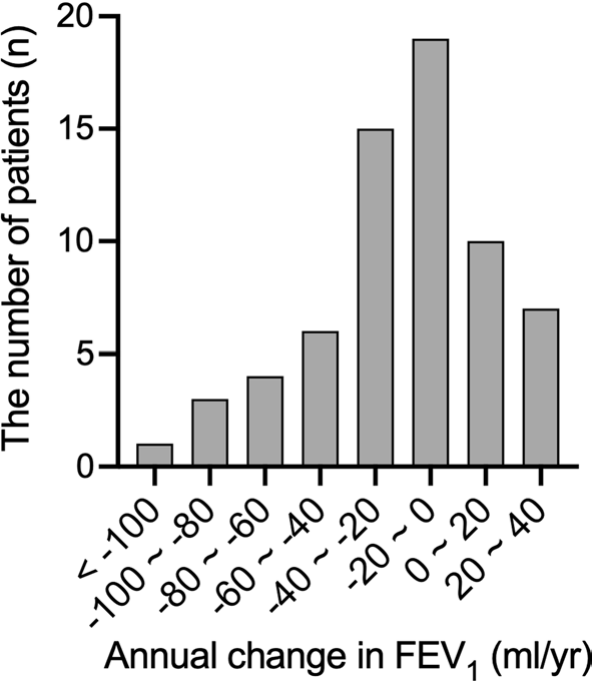
Distribution of annual change in FEV_1_ in patients with COPD_._ According to observation over 5 years, the annual change in FEV_1_ (mL) varied widely. The mean (SEM) was − 17.8 (4.0) mL/year (*n* = 65)

**Fig. 3:**
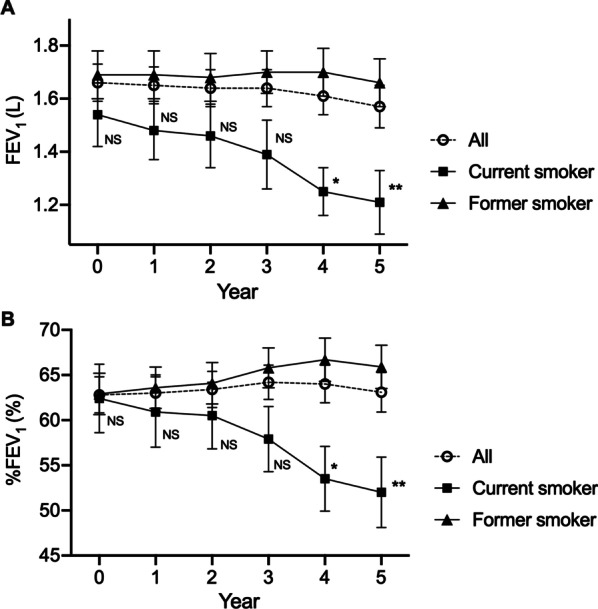
Comparison of FEV_1_ and %FEV_1_ over time between current and former smokers with COPD. There were significant differences in FEV_1_ (A) or %FEV_1_ (B) between current and former smokers both 4 and 5 years after baseline. The bars represent mean ± standard error of the mean. The differences between current and former smokers were analyzed using unpaired t-tests. NS: not significant, *: *p* < 0.05, ** *p* < 0.01

The annual change in FEV_1_ (mL/year) across 5 years varied widely, with a mean ± SEM of − 17.8 ± 4.0 and a range of − 142.0 to 34.0 (Fig. 2). Comparing changes over time in current and former smokers, FEV_1_ or %FEV_1_ were both significantly lower at 4 and 5 years after treatment in current smokers (Fig. 3). Comparisons between these cohorts are presented in Table 2. At baseline there was no significant difference in FEV_1_ between current and former smokers (*p* = 0.44) but 5 years after treatment it was lower in current smokers (*p* < 0.05). The annual changes in FEV_1_ and %FEV_1_ were significantly different between current and former smokers (− 66.2 mL/year vs. − 5.7 mL/year; *p* < 0.01 and − 2.1 mL/year vs. 0.6 mL/year; *p* < 0.01, respectively). No IOS parameter differed between current and former smokers (Table 2).

**Table 2:** Comparison of annual change in FEV_1_ between patients with COPD classified based on smoking status at baseline and comorbidity during the 5-year follow-up period

	Smoking status		Pneumonia		Asthma		Cancer		Cardio-vascular disease	
	Current	Former	+	−	+	−	+	−	+	−
Number of participants	13	52	25	40	24	41	18	47	17	48
Age	66.5 (2.9)	70.4 (1.0)	71.1 (1.7)	68.7 (1.2)	70.0 (2.0)	69.0 (1.1)	72.8 (1.6)	68.5 (1.2)	72.4 (1.4)	68.7 (1.2)
Sex; Male:Female	9:4	44:8	19:6	34:6	18:6	35:6	16:2	37:10	15:2	40:8
Body mass index	22.1 (0.4)	22.5 (0.4)	22.7 (0.6)	22.3 (0.5)	22.9 (0.7)	22.2 (0.4)	23.4 (0.8)	22.1 (0.4)	21.9 (0.8)	22.6 (0.4)
Smoking history (pack-years)	49.4 (5.2)	52.3 (2.0)	50.5 (3.0)	52.5 (2.4)	46.4 (1.4)	54.9 (2.8)	49.8 (2.1)	52.5 (2.5)	55.9 (4.7)	50.3 (1.9)
Current: Former			8:17	4:36	10:14	3:38**	3:15	10:37	3:14	10:38
*Laboratory values*										
Neutrophil in blood (count/µl)	4152 (285)	3740 (150)	4166 (220)	3594 (159)*	4238 (208)	3579 (163)*	4367 (237)	3614 (151)*	3688 (283)	3870 (151)
Eosinophil in blood (count/µl)	220.9 (34.0)	200.8 (18.0)	227.3 (25.1)	189.7 (20.3)	248.2 (29.4)	179.4 (17.4)*	220.5 (40.4)	198.7 (15.8)	169.2 (27.0)	217.4 (19.0)
Total IgE in serum (IU/ml)	392.7 (251.7)	417.2 (120.0)	468.4 (224.6)	374.9 (177.6)	686.6 (252.4)	179.4 (78.4)	680.1 (297.9)	309.7 (184.4)	451.6 (171.0)	398.4 (133.3)
mMRC	1.2 (0.1)	1.3 (0.1)	1.5 (0.1)	1.1 (0.1)**	1.4 (0.1)	1.2 (0.1)	1.4 (0.1)	1.2 (0.1)	1.3 (0.1)	1.3 (0.1)
CAT	7.3 (1.9)	5.6 (0.6)	7.3 (1.1)	5.1 (0.6)	6.9 (1.3)	5.4 (0.6)	7.7 (1.5)	5.3 (0.6)	5.4 (0.7)	6.2 (0.8)
*FEV1*										
Baseline (L)	1.54 (0.12)	1.69 (0.09)	1.41 (0.09)	1.83 (0.10)**	1.46 (0.09)	1.78 (0.10)*	1.46 (0.09)	1.74 (0.09)	1.53 (0.13)	1.71(0.09)
After 5 years (L)	1.21 (0.12)	1.66 (0.09)*	1.25 (0.08)	1.79 (0.10)**	1.31 (0.10)	1.72 (0.10)**	1.36 (0.10)	1.65 (0.10)	1.45 (0.12)	1.61 (0.09)
Annual change (ml/year)	− 66.2 (7.6)	− 5.7 (2.8)**	− 31.5 (7.8)	− 8.9 (3.7)**	− 30.2 (8.0)	− 10.8 (4.0)*	− 19.9 (8.8)	− 17.2 (4.5)	− 14.6 (0.4)	− 19.1 (5.0)
*%FEV1*										
Baseline (%)	62.4 (3.8)	62.9 (2.3)	58.3 (3.5)	65.8 (2.3)	57.7 (2.6)	65.8 (2.7)*	58.0 (2.9)	64.6 (2.5)	58.8 (4.5)	64.2 (2.2)
After 5 years (%)	52.0 (3.9)	65.9 (2.4)**	55.8 (3.6)	68.0 (2.4)**	55.0 (2.9)	67.9 (2.7)**	57.8 (33.3)	65.2 (2.7)	60.1 (4.6)	64.2 (2.4)
Annual change (%/year)	− 2.1 (0.20)	0.6 (0.12)**	− 0.49 (0.35)	0.44 (0.17)**	− 0.53 (0.37)	0.42 (0.16)**	− 0.05 (0.40)	0.11 (0.19)	0.26 (0.24)	0.00 (0.23)
*IOS*										
R5 (kPa/L/s)	0.32 (0.01)	0.28 (0.01)	0.33 (0.03)	0.26 (0.01)**	0.33 (0.02)	0.26 (0.01)**	0.29 (0.02)	0.29 (0.01)	0.28 (0.02)	0.29 (0.02)
R20 ( kPa/L/s)	0.24 (0.02)	0.20 (0.01)	0.22 (0.01)	0.30 (0.01)	0.23 (0.01)	0.20 (0.01)*	0.20 (0.01)	0.21 (0.01)	0.19 (0.01)	0.21 (0.01)
R5-R20 ( kPa/L/s)	0.09 (0.01)	0.08 (0.01)	0.11 (0.01)	0.06 (0.01)**	0.11 (0.01)	0.07 (0.01)*	0.09 (0.02)	0.08 (0.01)	0.09 (0.02)	0.08 (0.01)
X5 (kPa/L/s)	− 0.12 (0.01)	− 0.13 (0.01)	− 0.15 (0.02)	− 0.11 (0.01)*	− 0.15 (0.02)	− 0.12 (0.01)	− 0.13 (0.02)	− 0.13 (0.01)	− 0.13 (0.02)	− 0.13 (0.01)
Fres (Hz)	20.3 (1.4)	18.3 (0.8)	21.4 (1.0)	16.5 (0.8)**	20.7 (1.0)	17.5 (1.0)*	19.1 (1.4)	18.5 (0.9)	17.8 1.6)	19.0 (1.8)
AX (kPa/L)	0.99 (0.17)	0.90 (0.13)	1.27 (0.20)	0.64 (0.09)**	1.21 (0.18)	0.75 (0.13)*	0.95 (0.22)	0.92 (0.12)	0.97 (0.21)	0.90 (0.12)

The data are shown as mean (standard error of the mean) or number ratio. The differences between each pair were analyzed by unpaired t-tests or the chi-squared test

^*^p < 0.05

^**^p < 0.01

### Characteristics, the annual change in FEV_1_, and IOS in patients with and without comorbidities

Table 2 describes the characteristics of patients with and without each COPD comorbidity. There were no differences in age, sex, BMI, smoking history, total IgE, and CAT in the groups defined by presence or absence of any of the comorbidities. Patients with pneumonia, asthma, or cancer had higher neutrophil counts than those without each disease respectively. The asthmatic group included higher numbers of current smokers and had higher eosinophil counts than the non-asthmatic group (10:14 vs. 3:38; *p* < 0.01 and 248.2 vs. 179.4; *p* < 0.05, respectively). In the patients with pneumonia, the mMRC was higher than in those without pneumonia (1.5 vs. 1.1, *p* < 0.05). CAT scores did not differ between any group.

**Fig. 4:**
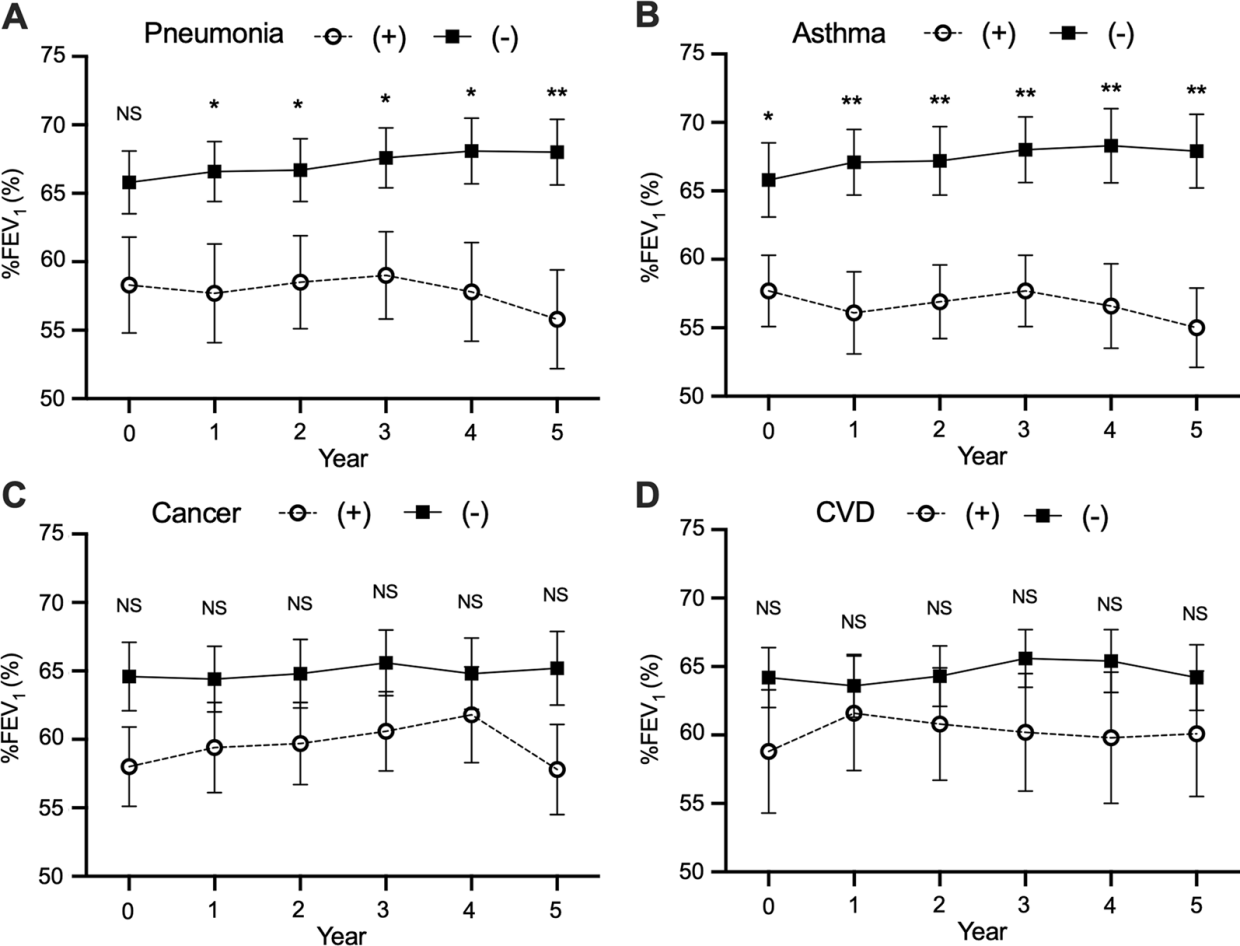
Comparison of %FEV_1_ in COPD patients with and without comorbidities. There were significant differences in %FEV_1_ in patients with and without pneumonia or asthma but not cancer or cardiovascular disease. The bars represent mean ± standard error of the mean. The differences between patients with and without each comorbidity were analyzed using unpaired t-tests. NS: not significant, *: *p* < 0.05, ** *p* < 0.01

At almost every annual timepoint, FEV_1_ and %FEV_1_ were significantly lower in patients with pneumonia or asthma than those without, but there were no differences in patients with or without cancer or cardiovascular disease (Fig. 4). There was significantly more annual decline in FEV_1_ in the pneumonia group than in the non-pneumonia group (− 31.5 vs. − 8.9, *p* < 0.01) and in the asthma group than in the non-asthma group (− 30.2 vs. − 10.8, *p* < 0.05). The annual decline in %FEV_1_ was notable in patients with pneumonia (− 0.49 vs. 0.44 in patients without pneumonia, *p* < 0.01) and asthma (− 0.53 vs. 0.42 in patients without asthma, *p* < 0.01). However, there was no change in annual decline in FEV1 and %FEV_1_ in patients with cancer and cardiovascular disease (Table 2).

Similar trends according to comorbidity were seen in IOS. Patients with pneumonia or asthma had higher R5, R5–R20, Fres, and AX values than those without, while the presence or absence of cancer or cardiovascular disease did not have a significant effect on the IOS results.

### Characteristics, the annual change in FEV_1_, and the rate of comorbidity in groups classified by IOS parameters

COPD patients were divided into high or low groups for each baseline IOS parameter and the differences in baseline characteristics, annual decline in FEV_1_ (%FEV_1_), and comorbidity rates compared (Table 3). The results of logistic regression analysis are shown in Additional file 1: Table S1. Notably, similar results were obtained.

**Table 3:** Comparison of annual change in FEV1 and comorbidity between patients with COPD classified by baseline impulse oscillometry system (IOS) parameter values

	R5		R20		R5-R20		X5		Fres		AX	
	≥ 0.39	< 0.39	≥ 0.27	< 0.27	≥ 0.10	< 0.10	≦ − 0.13	> − 0.13	≥ 17.7	< 17.7	≥ 0.55	< 0.55
Number of participants	11	54	8	57	21	44	26	39	38	27	36	29
Age	74.6 (1.8)	68.6 (1.1)*	70.8 (3.4)	69.5 (1.0)	72.1 (2.0)	68.5 (1.1)	72.7 (1.4)	67.6 (1.3)	70.6 (1.4)	68.3 (1.3)	71.1 (1.5)	67.9 (1.2)
Sex; Male:Female	6:5	47:7*	5:3	48:9	15:6	38:6	20:6	33:6	29:9	24:3	27:9	26:3
Body mass index	21.4 (1.0)	22.7 (0.4)	22.2 (1.4)	22.5 (0.4)	21.4 (0.7)	22.9 (0.5)	22.0 (0.6)	22.7 (0.5)	22.2 (0.5)	22.8 (0.5)	22.3 (0.6)	22.7 (0.5)
Smoking history (pack-years)	51.4 (2.4)	51.8 (2.2)	49.4 (2.3)	52.1 (2.0)	52.6 (3.2)	51.3 (2.3)	52.7 (1.6)	51.1 (2.9)	51.7 (2.0)	51.8 (3.5)	52.3 (2.6)	51.1 (2.7)
Current: Former	2:9	11:43	4:4	9:48	5:16	8:36	6:20	7:32	10:28	3:24	10:26	3:26
*Laboratory values*												
Neutrophil in blood (count/µl)	4253 (373)	3735 (140)	4445 (344)	3735 (129)	3984 (255)	3746 (155)	4043 (219)	3676 (166)	4036 (177)	3522 (191)	4038 (178)	3555 (192)
Eosinophil in blood (count/µl)	185 (36)	208 (17)	231 (48)	201 (18)	176 (20)	218 (21)	212 (23)	200 (21)	215 (20)	189 (25)	221 (22)	184 (22)
Total IgE in serum (IU/ml)	184 (91)	458 (127)	528 (410)	396 (109)	221 (106)	503 (149)	342 (153)	458 (148)	448 (166)	361 (113)	448 (175)	367 (105)
mMRC	1.6 (0.2)	1.2 (0.1)	1.4 (0.2)	1.3 (0.1)	1.5 (0.1)	1.2 (0.1)*	1.5 (0.1)	1.2 (0.1)*	1.4 (0.1)	1.1 (0.1)**	1.4 (0.1)	1.1 (0.1)*
CAT	8.5 (1,6)	5.4 (0.6)	5.9 (2.1)	6.0 (0.6)	7.4 (1.1)	5.3 (0.7)	7.5 (1.1)	4.9 (0.6)*	7.3 (0.9)	4.1 (0.6)**	6.6 (0.9)	5.1 (0.8)
*FEV1*												
Baseline (L)	1.04 (0.07)	1.79 (0.08)**	1.22 (0.10)	1.72 (0.08)*	1.17 (0.06)	1.89 (0.09)**	1.28 (0.07)	1.91 (0.09)**	1.34 (0.07)	2.11 (0.10)**	1.34 (0.06)	2.07 (0.11)**
After 5 years (L)	0.98 (0.08)	1.69 (0.08)**	1.04 (0.13)	1.65 (0.08)**	1.09 (0.05)	1.80 (0.09)**	1.16 (0.07)	1.85 (0.09)**	1.24 (0.07)	2.04 (0.10)**	1.22 (0.06)	2.00 (0.11)**
Annual change (ml/year)	− 12.7 (8.8)	− 19.0 (4.5)	− 36.8 (11.0)	− 15.3 (4.2)	− 17.1 (6.7)	− 18.3 (5.0)	− 24.2 (7.7)	− 13.8 (4.3)	− 20.2 (5.9)	− 14.8 (4.9)	− 22.1 (6.1)	− 12.8 (4.8)*
*%FEV1*												
Baseline (%)	50.1 (2.5)	65.4 (2.2)**	53.4 (3.9)	64.1 (2.1)	50.0 (2.7)	68.9 (2.1)**	54.0 (2.8)	68.7 (2.3)**	54.3 (2.0)	74.7 (2.0)**	54.6 (2.0)	73.0 (2.6)**
After 5 years (%)	50.1 (2.7)	65.8 (2.4)**	48.1 (2.7)	65.2 (2.3)**	50.1 (2.9)	69.3 (23)**	52.2 (2.8)	70.4 (2.5)**	53.6 (2.2)	76.6 (5.4)**	53.5 (2.1)	75.0 (2.8)**
Annual change (%/year)	0.001 (0.50)	0.08	− 1.05 (0.54)	0.22 (0.18)*	0.03 (0.29)	0.08 (0.23)	− 0.35 (0.35)	0.34 (0.18)	− 0.16 (0.32)	0.38 (0.19)	− 0.20 (0.28)	0.40 (0.19)*
*Comorbidity*												
Pneumonia n (+ : −)	7:4	19:35	5:3	19:38	13:8	13:31*	15:11	11:28*	20:18	6:21*	19:17	7:22*
Asthma n (+ : −)	7:4	17:37	6:2	18:3*	11:10	13:31	14:12	10:29*	19:19	5:22*	20:16	4:25**
Cancer n (+ : −)	3:8	15:39	1:7	17:40	7:14	11:33	8:18	10:29	11:27	7:20	11:25	7:22
Cardio-vascular dis. n (+ : −)	3:8	14:30	1:7	16:41	8:13	9:35	7:19	10:29	8:30	9:18	9:27	8:21

The data are shown as mean (standard error of the mean) or number ratio. The differences between each pair were analyzed by unpaired t-tests or the chi-squared test

^*^p < 0.05

^**^p < 0.01

There was no significant difference in any baseline characteristic based on IOS parameters, except that the high R5 group had a higher age (74.6 vs. 68.6, *p* < 0.05) and higher proportion of males (6:5 vs. 47:7, *p* < 0.05). The mMRC of the high R5–R20 group, the low X5 group, the high Fres group, and the high AX group was significantly higher than the corresponding other group in each case (1.5 vs. 1.2, *p* < 0.05; 1.5 vs. 1.2, *p* < 0.05; 1.4 vs. 1.1, *p* < 0.01; 1.4 vs. 1.1, *p* < 0.05, respectively). The CAT score was significantly higher only in the low X5 group and the high Fres group (7.5 vs. 4.9, *p* < 0.05, 7.3 vs. 4.3 *p* < 0.01, respectively).

For the annual decline in FEV_1_ and %FEV_1_, only the high AX group was significantly lower than the low AX group (− 22.1 vs. − 12.8, *p* < 0.05 and − 0.20 vs. 0.40, *p* < 0.05, respectively).

The ratios of each comorbidity were also compared between the groups classified by IOS values. The high R5–R20 group, the low X5 group, the high Fres group, and the high AX group had significantly higher numbers of patients with pneumonia than the corresponding other group (13:8 vs. 13:31, *p* < 0.05; 15:11 vs. 11:28, *p* < 0.05; 20:18 vs. 6:21, *p* < 0.05; 19:17 vs. 7:22, *p* < 0.05, respectively). For asthma, significant differences were observed between the groups based on R20 (6:2 vs. 18:39, *p* < 0.05), X5 (14:12 vs. 10:29, *p* < 0.05), Fres (19:19 vs. 5:22, *p* < 0.05), and AX (20:16 vs. 4:25, *p* < 0.01). There were no differences in the ratios of patients with cancer or cardiovascular disease between the groups defined by any IOS parameter.

## Discussion

In this observational study, we demonstrated that smoking cessation with proper treatment could prevent decline in FEV_1_, that pneumonia and asthma as comorbidities might result in decline in lung function, and that the IOS parameter AX at baseline can predict future decline in FEV_1_ in patients with COPD.

Recent studies have shown that the rate of decline in FEV_1_ in properly treated patients with COPD varies widely from rapid to relatively modest decline in lung function over a 3–5 year period [5, 35]. Because COPD patients who continue to smoke are at increased risk of marked progression compared with former smokers, smoking cessation is considered to be the most important tool in secondary prevention of FEV_1_ decline. Many studies have compared FEV_1_ decline between smokers and former smokers [2, 5, 36, 37]. In the present study, FEV_1_ in current smokers on LAMA and LABA treatment was reduced by 66.2 mL (2.1%) per year, far more than for former smokers, in which it was reduced by 5.7 ml (+ 0.6%) per year. Since 80% of patients were former smokers, the overall FEV_1_ reduction rate was relatively low at − 17.8 mL (+ 0.06%) per year. Change in respiratory function is considered to be suppressed by smoking cessation and appropriate treatment, which could improve the prognosis of patients with COPD. On the other hand, when IOS parameters were compared between current and former smokers, there were no differences, in agreement with a previous study [27]. Overall, in the present study smoking cessation and COPD therapy improved the prognosis of respiratory function as in previous studies, but could not be simply detected by IOS.

COPD often has various comorbidities related to smoking and aging, such as pneumonia, asthma, cancer, and cardiovascular diseases, and these negatively affect QOL and prognosis. Patients with a high frequency of exacerbations have a greater decline in FEV_1_ than those with a low frequency of exacerbations [9], and one cause of exacerbation is pneumonia. With regard to exacerbation, similar results were obtained (Additional file 1: Table S1). However, few studies have directly shown that pneumonia is associated with annual decline in FEV_1_. Our data showed that FEV_1_ decline in patients with pneumonia was higher than those without pneumonia (− 31.5 mL/year vs. − 8.9 mL/year). This indicates that the prognosis for COPD patients with episodes of pneumonia may be poor. The link between COPD and pneumonia may be explained by airway inflammation caused by a viral or bacterial infection.

Regarding ACO, the prognosis is controversial [10, 11, 38], so we examined changes in respiratory function over 5 years in COPD patients with or without asthma. We found that the %FEV1 changed more in patients with ACO than in those with COPD alone; the annual decline in FEV_1_ (%FEV_1_) in ACO was − 30 mL (− 0.53%) per year, compared with − 10.8 mL (0.42%) / year in COPD. This suggested that the prognosis for ACO could be poorer than that for COPD. Asthma-related airway inflammation in addition to COPD airway obstruction could have contributed to the decline in respiratory function.

Cancers other than lung cancer are often associated with COPD in the real world. However, while many studies report a strong relationship between reduction in FEV_1_ and the risk of lung cancer [39], there are few studies on the relationship between other cancers and FEV1 decline in COPD. In the present study, we found no differences in %FEV1 over 5 years or annual change in FEV1 in patients with and without cancer. The subset of cases with lung cancer was too small to analyze separately, and it remains a possibility that there would be a significant difference in FEV1 in this cohort. This is a topic for future study.

Regarding cardiovascular disease, low FEV1 and airflow obstruction are associated with atrial fibrillation [14], coronary heart disease [40], heart failure [41], and stroke [42]. However, there have been few studies on whether cardiovascular diseases are associated with the annual decline in FEV1 in COPD patients. In this study, we found no significant difference in the decline of FEV1 between patients with or without cardiovascular disease. In terms of the combined effect of cardiovascular disease and cancer on COPD, the annual decline in respiratory function should be more fully investigated in future.

IOS can determine the mechanical properties of the lung and differentiate between large and small airway obstruction. It seems to be more sensitive and detect changes in lung function earlier than spirometry in COPD patients [43]. The IOS parameters R5, R5–R20, and X5 (and not R20) were shown to correlate with FEV_1_ in a cohort of COPD patients, and over 1 year the changes in X5 correlated with the changes in FEV_1_ [25]. Peripheral measurement by IOS (R5–R20 and X5) correlates with the SGRQ and mMRC scores [29]. Although there are no defined reference values for COPD, pathologically abnormal IOS cutoffs have been proposed [27, 28]. Franz et. al. described subjects reporting respiratory symptoms with differing lung mechanics as measured by IOS, and in whom IOS had the potential to detect COPD pathology earlier than spirometry, and found that cutoff values in those with symptoms and meeting GOLD criteria were as follows: R20 = 0.39, R20 = 0.27, R5–R20 = 0.10, X5 =  − 0.13, and AX = 0.55 [30]. Separately, the cutoff value for Fres optimal to diagnose airflow obstruction in COPD patients was shown to be 17.7 [31]. As described in the methods, we classified the 65 COPD patients into two groups based on the above IOS cutoff values in the present study. To investigate the difference between inhaler treatments at baseline, the participants were classified into two groups according to the IOS value. Patients who had values more than the cutoff values had used more drugs, which indicates that patients with severe COPD tended to need stronger treatment (Additional file 1: Table S2). The annual change of FEV_1_ was compared in each pair. In these groups classified according to IOS, COPD patients with high AX at baseline had significantly greater annual decline in FEV_1_ than patients with low AX (*p* < 0.05), indicating a correlation between AX value and annual change in FEV_1_ over 5 years. The group with decreased %FEV_1_ (n = 25) had significantly higher AX than the group with increased %FEV_1_ (n = 40), with an odds ratio of 4.19 and 95% confidence period of 1.26–15.71 (Additional file 1: Table S1). Similar results were also obtained when compared between the amount of change in AX and that in FEV_1_ decline (r = 0.28, *p* = 0.013, Spearman’s correlation analysis). R5–R20, X5, and Fres did not correlate with deterioration of respiratory function. It is known that R5–R20 reflects obstruction in the distal airways [44] and X5 reflects the elastic components of the lung. On the other hand, AX reflects changes in peripheral airway obstruction and reduction of lung compliance, which are typically observed in COPD [22, 45]. This may be why only AX was significantly associated with FEV_1_ decline.

The current study had some limitations. First, the study population was small, and the study design was retrospective. To consider potential confounding factors and covariates such as smoking status, exacerbation, pneumonia, and high AX, logistic regression analysis was performed (Additional file 1: Table S1). Notably, very similar results were obtained. However, because this was a small cohort study, it was difficult to judge whether the results were statistically significant and multivariate analysis could not be performed. We hope to clarify this in future research. Second, 32 of the 97 patients were excluded due to treatment interruption or hospital transfer. This is unlikely to have introduced bias, as there was no difference in the background characteristics of these patients compared to the 65 patients included in the study (data not shown). COPD involves various factors and is difficult to analyze simply. Although COPD treatment changes over time depending on the stage, risk of exacerbation, and symptoms, we did not consider whether the treatment method is related to the decrease in FEV_1_. Third, since IOS is an emerging technique, the exact meaning and interpretation of its parameters are limited. Reference values in different populations have not yet been established. However, pathologically abnormal values were proposed for COPD patients in previous studies [30, 31] and we used these to investigate the decline in FEV_1_.

## Conclusion

This study showed that smoking cessation in addition to proper treatment was able to slow FEV_1_ decline in patients with COPD. Coexistence of pneumonia or asthma was associated with FEV_1_ decline, but cancer and cardiovascular disease were not. Thus, continuing smoking, complications in pneumonia and asthma would be risk factors for the progression of COPD. Our data suggest that baseline AX, which may detect and evaluate small airways and emphysema simultaneously, was the IOS parameter most related to the annual decline in FEV_1_ for COPD and may accurately predict prognosis in patients with COPD.

## Supplementary Information

**Additional file1: Table S1.** Odds ratio for COPD patients with and without decline in FEV1 using the logistic regression analysis. *: p < 0.05, **: p < 0.01. **Table S2.** Comparison of single- or multiple-inhaler therapy for COPD (single, double or triple therapy) at baseline between groups based on IOS values. *: p < 0.05, **: p < 0.01 (Pearson's chi-squared test).

## Data Availability

The data that support the findings of this study are available on request from the corresponding author on reasonable request.

## Abbreviations

ACO: Asthma–COPD overlap
mMRC: Modified British Research Council scale
CAT: COPD Assessment Test
LAMA: Long acting muscarinic antagonist
LABA: Long acting β2 agonist
ICS: Inhaled corticosteroid
FVC: Forced vital capacity
FEV_1_: Forced expiratory volume in one second
MMEF: Maximum mid-expiratory flow
PEF: Peak expiratory flow
IOS: Impulse oscillometry system
R5: Resistance at 5 Hz
R20: Resistance at 20 Hz
R5 − R20: The difference between R5 and R20
X5: Reactance at 5 Hz
Fres: Resonance frequency
AX: Area of low-frequency reactance
