# Supplementary material for: Association between annual change in FEV1 and comorbidities or impulse oscillometry in chronic obstructive pulmonary disease
Source: BMC Pulm Med. 2022 May 8;22:185. doi: 10.1186/s12890-022-01980-6 (PMC9080138; doi:10.1186/s12890-022-01980-6)
Supplement: Supplementary file 1 — Additional file1: Table S1. Odds ratio for COPD patients with and without decline in FEV1 using the logistic regression analysis. *: p < 0.05, **: p < 0.01. Table S2. Comparison of single- or multiple-inhaler therapy for COPD (single, double or triple therapy) at baseline between groups based on IOS values. *: p < 0.05, **: p < 0.01 (Pearson's chi-squared test). [file 12890_2022_1980_MOESM1_ESM.doc]

**ADDITIONAL FILE**

**Association between annual change in FEV_1_ and comorbidities or impulse oscillometry in chronic obstructive pulmonary disease**

Hiroyuki Sugawara, Atsushi Saito, Saori Yokoyama, Kazunori Tsunematsu,

and Hirofumi Chiba

**Table S1.**

**Table S2.**

**RESULTS**

**Table S1**. Odds ratio for COPD patients with and without decline in FEV1 using the logistic regression analysis. *: p < 0.05, **: p < 0.01

**Table S2**. Comparison of single- or multiple-inhaler therapy for COPD (single, double or triple therapy) at baseline between groups based on IOS values. *: p < 0.05, **: p < 0.01 (Pearson's chi-squared test).

| Value of IOS | | <= cut-off | | | cut-off < | | |  |
| --- | --- | --- | --- | --- | --- | --- | --- | --- |
| Inhaler Tx | | Single | Double | Triple | Single | Double | Triple | p |
| IOS parameter | R5 | 0 | 1 | 10 | 18 | 15 | 21 | * |
|  | R20 | 0 | 2 | 6 | 18 | 14 | 25 | NS |
|  | R5-R20 | 1 | 2 | 18 | 17 | 13 | 14 | ** |
|  | X5 | 1 | 5 | 20 | 17 | 11 | 11 | ** |
|  | Fres | 2 | 9 | 27 | 16 | 7 | 4 | ** |
|  | AX | 3 | 7 | 26 | 15 | 8 | 6 | ** |
